# Using a Bayesian workflow approach to define the optimal level of anatomy/functionality voxels clustering for the prediction of tau propagation and cortical loss in Alzheimer's disease

**DOI:** 10.1002/alz.093845

**Published:** 2025-01-09

**Authors:** Jean‐Paul Soucy, Clyde J Belasso, Zhengchen Cai, Gleb Bezgin, Jenna Stevenson, Nesrine Rahmouni, Cécile Tissot, Firoza Z Lussier, Pedro Rosa‐Neto, Hassan J Rivaz, Habib Benali

**Affiliations:** ^1^ Montreal Neurological Institute, McGill University, Montréal, QC Canada; ^2^ Concordia University, Montreal, QC Canada; ^3^ McGill University, Montreal, QC Canada; ^4^ McGill Centre for Studies in Aging, Department of Neurology and Neurosurgery, McGill University, Montreal, QC Canada; ^5^ McGill University Research Centre for Studies in Aging, Montreal, QC Canada; ^6^ The McGill University Research Centre for Studies in Aging, Montreal, QC Canada; ^7^ Translational Neuroimaging Laboratory, The McGill University Research Centre for Studies in Aging, Montréal, QC Canada

## Abstract

**Background:**

Tau aggregates in Alzheimer’s disease (AD) induce loss of synapses and neurons, leading to cognitive impairment. Predicting tau and neurodegeneration temporal evolution could be used for prognostication and for assessing results of therapeutic trials. Tau PET and MRI volumetry are reliable markers of disease stage, but cost and radiation protection considerations limit research measurement frequency, lowering the accuracy of disease progression modeling. Here, we evaluate, using Bayesian analysis, whether models based on limited numbers of observations can be refined to better predict the temporal trajectory of pathology.

**Method:**

Imaging data comes from subjects (113; 68 females; 18 AD dementia, 23 MCI and 72 cognitively normal) of the TRIAD cohort (McGill University) who have been evaluated at least twice ( 1 year interval) with both tau PET ([18F]MK‐6240) and structural MRI. Four probability models were evaluated: 1‐ a basic one, assuming that all data points come from 1 data distribution; 2‐ one where subjects' observations are clustered within anatomical ROIs, where an independent distribution is hypothesized; 3‐ data is clustered within known physiological networks, each networks’ distribution parameters having their own specific values ; 4‐ a model assuming that subjects’ observations are described by a distribution of voxel parameters dictated by both the ROI and network(s) in which they lay. Bayesian data analysis was used to compare the predictive accuracy of those models for progression at 1 year from baseline of tau PET and MRI data.

**Result:**

Model 4 was the most accurate model for both tau and cortical thickness prediction. We therefore used it to perform posterior predictions across hemispheres, showing that the prediction curves of the left and right hemispheres for the pericalcarine cortex differ. We also noticed a decreasing trend in the CN tau curve for the left hemisphere as the rate of cortical thinning increases. In contrast, there is an increasing trend in the AD tau curve as the rate of cortical thinning increases.

**Conclusion:**

The model that incorporated both ROI‐level and network‐level information was the best predictor of progression, and such an approach can reveal underappreciated properties of the disease (i.e., laterality).

